# A community-oriented survey on the association between androgenetic alopecia and metabolic syndrome in Chinese people

**DOI:** 10.3389/fmed.2022.1009578

**Published:** 2022-11-10

**Authors:** Hongliu Zhu, Haijian Guo, Yihong Gao, Yuegang Wei, Tao Mao, Jianqiu Yang

**Affiliations:** ^1^Department of Dermatology, Jiangyin Hospital of Traditional Chinese Medicine, Wuxi, China; ^2^Integrated Business Management Office, Jiangsu Provincial Center for Disease Control and Prevention, Nanjing, China; ^3^Department of Dermatology, The Affiliated Hospital of Nanjing University of Chinese Medicine, Nanjing, China; ^4^Department of Health Education, Jiangsu Provincial Center for Disease Control and Prevention, Nanjing, China

**Keywords:** androgenetic alopecia, metabolic syndrome, systolic blood pressure, dyslipidemia, diabetes mellitus

## Abstract

**Background:**

Several studies on Caucasians have revealed a positive relationship between androgenetic alopecia (AGA) and metabolic syndrome (MS). However, this correlation varies in different contexts. Currently, the association of AGA with MS is yet to be studied and elucidated in Chinese people.

**Objective:**

To evaluate the association between AGA and MS in the Chinese population.

**Methods:**

This study included information on components of MS along with other possible risk factors in a total of 3,703 subjects. The patients’ loss of hair was assessed using Hamilton-Norwood and Ludwig classification method.

**Results:**

In this study, 29.88% of male and 27.58% of female AGA patients were diagnosed with MS, while the rest were regarded as controls (29.95% of male and 27.89% of female control subjects) (*P* > 0.05). The AGA males presented significantly higher systolic and diastolic blood pressure than the male control subjects (SP: *P* = 0.000; DP: *P* = 0.041). Among females with AGA, waist circumference, hip circumference, and waist-hip ratio elevated the loss of hair compared to that of the female controls (*P* = 0.000, *P* = 0.020, *P* = 0.001, respectively).

**Conclusion:**

Our study indicated no direct association between AGA and MS in Chinese people. However, a close relationship was observed between AGA and systolic blood pressure.

## Introduction

Androgenetic alopecia (AGA), also known as male pattern baldness, is a type of hair loss commonly prevailing in males. According to statistics, about 60–70% of the population is affected by AGA globally (1), affecting 21.6% of Chinese males and 6.0% of Chinese females adults (2). Epidemiological studies have revealed a positive association between AGA and metabolic syndrome (MS), with some studies labeling AGA as an independent risk factor for cardiovascular disease and MS (3). However, this correlation has been denied by some reports (4).

Contrastingly, most of the previous studies have established the relationship between AGA and MS in Caucasian subjects. However, evidence elucidating the correlation between AGA and MS in Chinese people remains insufficient.

Therefore, our study compared the presence of MS components in both male and female AGA patients with control subjects in a Chinese population.

## Participants and methods

### Participants

A population-oriented, cross-sectional study was implemented between July and September 2020. This study was based on a multistage, stratified sampling method and was initiated in Yandu and Jurong, two cities of Jiangsu Province. Next, two or three districts/towns were randomly chosen from these two cities separately. Of these, we randomly picked out eight neighborhoods/villages as the subjects. The eligibility of the study included people aged >18 years without any conditions of mental disorders, along with pregnant and lactating women. Our study enrolled a total of 3,703 participants, including 1,414 men and 2,289 women.

### Measurements

**FIGURE 1:**
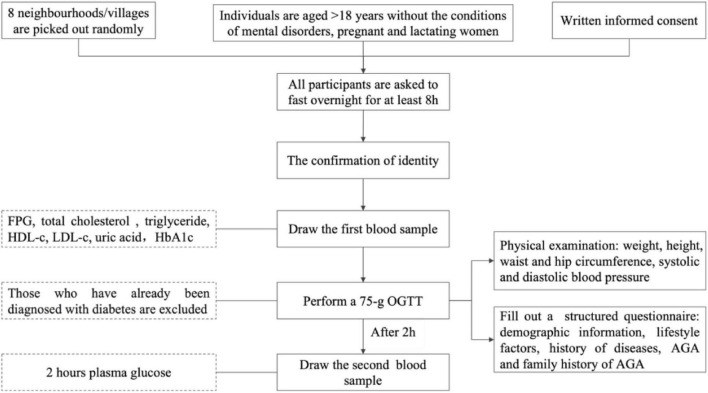
Flowchart of the study process.

Figure 1 depicts the flow chart of the study process. Before beginning the screening process, all participants were asked to fast overnight for at least 8 h, and the first blood sample was drawn after confirming their identity. Next, a 75-g OGTT was performed, and the second blood sample was drawn after 2 h. During the next 2 h, participants were measured for general non-laboratory parameters, including weight and height, waist and hip circumference, and systolic and diastolic blood pressure. The demographic information, lifestyle factors, and history of diseases were obtained using a structured questionnaire. In the same central laboratory (Adicon, Nanjing), venous blood sample tests were conducted using the same method. The third fingertip of the non-dominant hand was cleaned using 75% ethyl alcohol, and the skin was punctured immediately after drying. Subsequently, a second blood droplet was obtained by firmly squeezing the tested finger. AGA was diagnosed based on clinical findings and family history.

### Diagnostic criteria

The definition of MS was based on the criteria defined by the Diabetes Branch of the Chinese Medical Association. Subjects were required to have three or more of the following criteria: (i) BMI ≥ 25 kg/m^2^; (ii) fasting plasma glucose (FPG) levels ≥ 6.1 mmol/L and/or 2 h plasma glucose (2 h PG) ≥ 7.8 mmol/L, and/or treated diabetes; (iii) BP ≥ 140/90 mmHg and/or treated hypertension; (iv) serum TG ≥ 1.70 mmol/L and/or HDL-c <0.9 mmol/L. BMI was calculated by dividing weight (kg) by height (m^2^). A value of 18.5 ≤ BMI < 24.0 was considered normal, 24.0 ≤ BMI < 28.0 was overweight, and BMI ≥ 28.0 was obese. Similarly, the waist-hip ratio was calculated by dividing the waist circumference by the hip circumference (cm). Central obesity was considered if waist circumference was >90 cm in men and >85 cm in women. Hyperuricemia (HUA) was defined if serum uric acid (UA) levels were >420 μmol/L in male subjects and > 360 μmol/L in females.

Androgenetic alopecia was diagnosed based on family history and clinical findings, which included increased hair thinning pattern on the frontal/parietal scalp, with male patients showing greater hair density in the occipital region while female patients showed retention of the frontal hairline and also the presence of miniaturized hair. The hair loss assessment was performed by two dermatologists. The severity of AGA was determined using the Hamilton-Norwood scale for male and Ludwig scale for female patients.

Exclusion criteria included other types of alopecia, patients who received hormone replacement therapy using testosterone, the presence of hyperaldosteronism, and a known cause of hyperandrogenism.

### Statistical analysis

Epidata 3.1 dual computer was used to input all data, while SPSS 17.0 software was used for statistical analysis. Data were described using incidence, mean ± standard deviation, ANOVA, Chi-square test, and other methods. A value of *P* < 0.05 was considered statistically significant. Furthermore, a logistic regression model was employed to assess the associations between each possible risk factor (BMI, waist circumference, WHR, fasting plasma glucose, BP, and serum level of lipids) and the risk of AGA.

### Ethical statement

This study was conducted following the Declaration of Helsinki and was approved by the Ethics Review Committee of Jiangsu Provincial Centre for Disease Control and Prevention (JSJK2016-B003-03) and Zhongda Hospital, Southeast University. Also, each participant provided written informed consent.

## Results

We investigated 1,414 men, aged between 21 and 79 years, with an average age of 55.91 ± 8.91 years, and 2,289 women, aged between 22 and 85 years, with an average age of 54.95 ± 8.43 years. Age, BMI, central obesity, hypertension, pathoglycemia, dyslipidemia, and MS are summarized in Table 1. Among males, 646 subjects (45.72%) were diagnosed with AGA, and based on the Hamilton-Norwood scale, 10.22% belonged to stage I, 31.42% to stage II, 19.97% to stage III, 13.62% to stage IV, 11.30% to stage V, 7.28% to stage VI, and 6.19% to stage VII AGA. Of all female participants, 116 (5.05%) were diagnosed with AGA. Of these, 33.62% were in stage I, 52.59% in stage II, and 13.79% in stage III based on the Ludwig scale.

**TABLE 1:** Demographic characteristics of the subjects.

	Males (1,414)		Females (2,289)	
		
	Cases	Proportion (%)	Cases	Proportion (%)
**Age**				
<25	3	0.21	2	0.09
25–34	40	2.83	64	2.80
35–44	111	7.85	169	7.38
45–54	401	28.36	779	34.03
55–64	581	41.08	955	41.72
>65	278	19.66	320	14.00
**BMI**				
24.0 ≤ BMI < 28.0	602	42.57	922	40.28
BMI ≥ 28.0	300	21.22	420	18.35
Central obesity	540	38.19	865	37.79
Hypertension	661	46.75	1,117	48.80
**Pathoglycemia**				
Prediabetes	134	9.48	198	8.65
Diabetes	261	18.46	340	14.85
**Dyslipidemia**				
Total cholesterol	46	3.25	109	4.76
Triglyceride	276	19.52	367	16.03
HDL-c	108	7.64	56	2.45
LDL-c	16	1.13	34	1.49
MS	423	29.91	638	27.87
Hyperuricemia	308	21.78	74	3.23
AGA	646	45.72	116	5.05

Prediabetes: fasting blood glucose ≥ 6.1 mmol/L but <7.0 mmol/L, or 2 h PG ≥7.8 mmol/L but <11.1 mmol/L.

The control group consisted of non-AGA subjects (768 males and 2,173 females). Statistically significant differences were observed in the mean age between both groups (*P* = 0.000, Table 2). Moreover, systolic and diastolic blood pressure were significantly higher in males with AGA than those in the control group (*P* = 0.000, *P* < 0.05, respectively). However, other indicators showed no statistical differences. In female AGA patients, the values of waist circumference, hip circumference, and waist-hip ratio were significantly higher than those in the control group (*P* = 0.001, *P* = 0.003, *P* < 0.05, respectively). Systolic blood pressure and total cholesterol were also higher in AGA female patients compared to the control group, with statistically significant differences in all the above-mentioned factors.

**TABLE 2:** Comparison between the MS risk factors.

	Males		*P*-value	Females		*P*-value
			
	AGA (*n* = 646)	Controls (*n* = 768)		AGA (*n* = 116)	Controls (*n* = 2,173)	
Age	57.92 ± 7.50	54.20 ± 9.62	0.000*	58.21 ± 7.27	54.78 ± 8.45	0.000*
BMI	25.30 ± 3.38	25.67 ± 7.62	0.256	25.34 ± 3.45	25.18 ± 7.14	0.811
Waist circumference	87.38 ± 12.30	87.01 ± 9.94	0.542	85.44 ± 8.95	82.37 ± 9.57	0.001*
Hip circumference	95.93 ± 10.00	95.43 ± 6.94	0.267	96.08 ± 6.10	94.03 ± 7.34	0.003*
WHR	0.91 ± 0.05	0.91 ± 0.06	0.727	0.89 ± 0.06	0.88 ± 0.07	0.038*
SBP	136.36 ± 18.22	132.37 ± 17.75	0.000*	141.51 ± 21.21	136.18 ± 20.28	0.006*
DBP	86.78 ± 11.38	85.51 ± 11.82	0.041*	84.76 ± 12.07	83.96 ± 11.94	0.484
FPG	5.79 ± 1.23	5.89 ± 1.37	0.158	5.90 ± 1.77	5.69 ± 1.39	0.208
TC	4.45 ± 0.85	4.50 ± 0.89	0.322	4.89 ± 0.75	4.67 ± 0.89	0.008*
TG	1.81 ± 1.83	1.95 ± 2.35	0.230	1.70 ± 1.68	1.67 ± 1.34	0.815
HDL-c	1.40 ± 0.28	1.39 ± 0.30	0.537	1.50 ± 0.22	1.47 ± 0.27	0.234
UA level	358.24 ± 79.49	355.80 ± 85.62	0.582	283.43 ± 75.62	273.98 ± 64.79	0.129

*P < 0.05.

Further analysis was conducted considering the severity of AGA as the standard. Our results showed that male subjects with severe AGA (grade V–VII) indicated higher risks of hypertension, MS, and hyperuricemia compared to those with mild (grade I–II) and moderate AGA (grade III–IV) and the controls. However, only hypertension reached a significant statistical difference (Table 3). In females, the severity of AGA showed significantly elevated waist circumference, hip circumference, and waist-hip ratio compared to that of the control group (*P* = 0.000, *P* = 0.020, *P* = 0.001, respectively, Tables 4, 5). Generally, in both males and females with AGA, the severity of hair loss showed an increase in the incidence of hypertension, dysglycemia, dyslipidemia, MS, and hyperuricemia.

**TABLE 3:** Comparison of MS risk factors among men exhibiting different severity of AGA.

	AGA group (Norwood classification)			Control group (*n*, %)	χ^2^	*P*-value
	
	I–II (*n*, %)	III–IV (*n*, %)	V–VII (*n*, %)			
BMI ≥ 25	149 (55.39)	102 (47.00)	85 (53.13)	423 (55.08)	4.829	0.185
24.0 ≤ BMI < 28.0	125 (46.47)	86 (39.63)	71 (44.38)	320 (41.67)	2.908	0.406
BMI ≥ 28.0	56 (20.82)	41 (18.89)	34 (21.25)	169 (22.01)	1.102	0.798
Central obesity	112 (41.64)	75 (34.56)	58 (36.25)	295 (38.41)	2.834	0.418
Hypertension	122 (45.35)	119 (54.84)	91 (56.86)	329 (42.84)	17.223	0.001*
Pathoglycemia	117 (43.49)	82 (37.79)	73 (45.63)	329 (42.84)	2.756	0.431
Diabetes	47 (17.47)	33 (15.21)	29 (18.13)	152 (19.79)	2.617	0.455
Dyslipidemia	64 (23.79)	54 (24.88)	41 (25.63)	197 (25.65)	0.392	0.942
TC	10 (3.72)	7 (3.23)	2 (1.25)	27 (3.52)	2.393	0.495
TG	47 (17.47)	41 (18.89)	34 (21.25)	154 (20.05)	1.216	0.749
HDL-c	23 (8.55)	16 (7.37)	12 (7.50)	57 (7.42)	0.394	0.941
MS	80 (29.74)	60 (27.65)	53 (33.13)	230 (29.95)	1.322	0.724
HUA	57 (21.19)	46 (21.20)	38 (23.75)	167 (21.74)	0.463	0.927

*P < 0.05. Pathoglycemia includes pre-diabetes and diabetes; dyslipidemia refers to abnormal levels of total cholesterol, triglyceride, high-density lipoprotein, and low-density lipoprotein.

**TABLE 4:** Comparison of MS risk factors among women exhibiting different severity of AGA.

	AGA group (Ludwig classification)			Control group (*n*, %)	χ^2^	*P*-value
	
	I (*n*, %)	II (*n*, %)	III (*n*, %)			
BMI ≥ 25	39 (100)	61 (100)	16 (100)	2,172(94.89)	0.047	0.829
24.0 ≤ BMI < 28.0	14 (35.90)	29 (47.54)	8 (50.00)	871 (40.08)	1.422	0.233
BMI ≥ 28.0	2 (5.13)	14 (22.95)	5 (31.25)	399 (18.36)	0.724	0.395
Central obesity	12 (30.77)	36 (59.02)	13 (81.25)	804 (37.00)	20.198	0.000*
Hypertension	22 (56.41)	34 (55.74)	7 (43.75)	1,054(48.50)	0.796	0.372
Pathoglycemia	16 (41.03)	26 (42.62)	9 (56.25)	914 (42.06)	0.477	0.490
Diabetes	6 (15.38)	14 (22.95)	3 (18.75)	317 (14.59)	2.770	0.096
Dyslipidemia	7 (17.95)	9 (14.75)	3 (18.75)	446 (20.52)	1.083	0.298
TC	4 (10.26)	2 (3.28)	1 (6.25)	102 (4.69)	0.059	0.808
TG	4 (10.26)	9 (14.75)	2 (12.50)	352 (16.20)	0.552	0.457
HDL-c	0 (0.00)	0 (0.00)	0 (0.00)	56 (2.58)	2.684	0.101
MS	8 (20.51)	18 (29.51)	6 (37.50)	606 (27.89)	0.171	0.679
HUA	2 (5.13)	7 (11.48)	0 (0.00)	65 (2.99)	6.692	0.010*

*P < 0.05. Pathoglycemia includes pre-diabetes and diabetes; dyslipidemia refers to abnormal levels of total cholesterol, triglyceride, high-density lipoprotein, and low-density lipoprotein.

**TABLE 5:** Comparison of waist circumference, hip circumference, and waist-hip ratio values between women with different severity of AGA and the control group.

	AGA group (Ludwig classification)			Control group	*F*	*P*-value
	
	I	II	III			
Waist circumference	81.94 ± 7.54	86.52 ± 9.01	89.83 ± 9.08	82.37 ± 9.57	6.975	0.000*
Hip circumference	95.12 ± 5.49	96.38 ± 6.48	97.24 ± 6.01	94.03 ± 7.34	3.303	0.020*
WHR	0.86 ± 0.06	0.90 ± 0.06	0.922 ± 0.05	0.88 ± 0.07	5.442	0.001*

*P < 0.05.

Furthermore, we investigated the correlation of AGA with other relevant indicators of MS, where both dependent (AGA) and independent variables (BMI, abnormal blood glucose, hypertension, dyslipidemia, waist circumference, and hyperuricemia) were included in the ordered logistic regression model analysis.

Upon introducing independent variables, the results of the likelihood ratio test showed a statistical significance (male: χ^2^ = 17.583, *P* < 0.001; female: χ^2^ = 23.576, *P* < 0.01), suggesting their effectiveness on AGA.

Table 6 shows that the *P*-value of hypertension in male subjects was <0.001, while the *P*-values of BMI, abnormal blood glucose, dyslipidemia, waist circumference, and hyperuricemia were all greater than 0.05. This suggested that hypertension, instead of BMI, blood glucose, blood lipids, and other factors, affected AGA in males. Furthermore, the partial regression coefficient for hypertension was −0.412, indicating a significant positive relation between hypertension and the severity of AGA.

**TABLE 6:** Multivariate logistic regression analysis including the MS parameters of male AGA patients.

	Coefficients	SE	Wald χ^2^	*P*-value	95% confidence interval	
					
					Lower limit	Upper limit
**Dependent variables**						
Grade = 0	0.075	0.170	0.195	0.659	–0.259	0.409
Grade = 1	1.463	0.176	69.288	0.000	1.119	1.808
**Independent variables**						
BMI ≥ 25 = 0	0.202	0.132	2.336	0.126	–0.057	0.462
BMI ≥ 25 = 1						
Pathoglycemia = 0	0.049	0.107	0.210	0.647	–0.161	0.259
Pathoglycemia = 1						
Hypertension = 0	−0.412	0.106	15.228	0.000	–0.619	–0.205
Hypertension = 1						
Dyslipidemia = 0	−0.044	0.113	0.152	0.696	–0.265	0.177
Dyslipidemia = 1						
Waistline = 0	0.019	0.135	0.019	0.890	–0.246	0.284
Waistline = 1						
HUA = 0	0.027	0.130	0.045	0.833	–0.227	0.282
HUA = 1						

Table 7 shows that dyslipidemia, waist circumference, and hyperuricemia in female subjects showed corresponding *P*-values of <0.05. However, abnormal BMI, blood glucose, and high blood pressure parameters with *P*-values greater than 0.05 suggested that dyslipidemia, waist circumference, and hyperuricemia, rather than BMI, blood pressure, blood glucose, and other factors, affected AGA in women. Moreover, the partial regression coefficients of dyslipidemia, waist circumference, and hyperuricemia were found to be 0.508, −0.832, and −1.033, respectively, indicating their positive correlation with the severity of AGA.

**TABLE 7:** Multivariate logistic regression analysis, including the MS parameters of female AGA patients.

	Coefficients	SE	Wald χ^2^	*P*-value	95% confidence interval	
					
					Lower limit	Upper limit
**Dependent variables**						
Grade = 0	1.983	0.378	27.516	0.000	1.242	2.724
Grade = 1	2.515	0.385	42.736	0.000	1.761	3.268
**Independent variables**						
BMI ≥ 25 = 0	0.432	0.240	3.244	0.072	–0.038	0.902
BMI ≥ 25 = 1						
Pathoglycemia = 0	0.010	0.203	0.002	0.961	–0.388	0.408
Pathoglycemia = 1						
Hypertension = 0	−0.204	0.200	1.042	0.307	–0.596	0.188
Hypertension = 1						
Dyslipidemia = 0	0.508	0.227	5.020	0.025	0.064	0.953
Dyslipidemia = 1						
Waistline = 0	−0.832	0.241	11.886	0.001	–1.305	–0.359
Waistline = 1						
HUA = 0	−1.033	0.384	7.258	0.007	–1.785	–0.282
HUA = 1						

## Discussion

Androgenetic alopecia is the most common hair loss observed in both genders. Although the onset of AGA is not dependent on race, it varies based on it. Generally, the prevalence of AGA is highest in Caucasians, with 30 and 50% of white men having AGA by the age of 30 and 50 years, respectively (1). We found that the prevalence rate of AGA was 45.72% in Chinese males and 5.05% in Chinese females. Compared to the last decade (2), the onset rate of AGA has more than doubled in Chinese males but has remained nearly unchanged in females. Moreover, the prevalence of AGA has been showing a global increasing trend (5).

Metabolic syndrome is a series of metabolic disorders associated with an increased risk of cardiovascular disease (6). Several factors are considered responsible for the pathogenesis of MS, including genetic predisposition, insulin resistance, obesity, hypertension, dyslipidemia, vascular abnormalities, inflammation, hyperandrogenism, and hyperuricemia. Previous studies on Chinese people have shown the prevalence rate of MS to be 19.58–24.2% (7, 8) while our study showed the prevalence of MS to be 29.91% in males and 27.87% in females. Cotton et al. (3) was the first to argue regarding a certain implication of the risk of cardiovascular disease in male AGA patients. Since then, many studies have shown the association of AGA with a high risk of MS, with such patients showing a significantly worse metabolic profile (9). In our study, although AGA patients were at an advanced age, a positive correlation was not observed between AGA and MS. Our results only showed significantly higher systolic blood pressure levels in both male and female AGA patients compared to that of the control. Furthermore, some previous studies have revealed that AGA patients have higher blood pressure (4, 10, 11), which can be explained by the increase in salt corticosteroids (including aldosterone) (12). Moreover, studies have shown that compared to patients with hypertension in the control group, AGA patients with hypertension showed higher levels of aldosterone. Besides, the increase in serum aldosterone level may be directly involved in the development of AGA. Contrastingly, elevated aldosterone levels may lead to impaired vascular endothelial function, decreased vascular compliance, and promoted renal reabsorption of water and sodium ions, resulting in the retention of water and sodium and, in turn, hypertension. Noteworthily, spironolactone, an aldosterone receptor antagonist, whose main mechanism of action is anti-androgenicity, was also used in the treatment of female patients with AGA.

Our results showed a higher prevalence of central obesity in the female AGA group than in the control group, which was dependent on the severity of AGA. However, the female patients with AGA showed no statistical difference either in weight or BMI compared to the control. Arias-Santiago et al. (11) reported a higher mean circumference in female AGA patients compared to that of the control (86.5 vs. 37.8%, *P* = 0.001) but showed no difference in weight or BMI. This suggested an abdominal redistribution of fat in female AGA patients, which was an important risk factor for cardiovascular disease.

## Conclusion

Although many studies have observed an association between AGA and MS, it has not been elucidated yet. Even in our study, we did not observe a relationship between AGA and MS. However, an early diagnosis remains a non-negligible issue since significantly elevated systolic blood pressure level is a risk factor for cardiovascular disease.

## Data availability statement

The original contributions presented in the study are included in the article/supplementary material, further inquiries can be directed to the corresponding author.

## Ethics statement

The studies involving human participants were reviewed and approved by the Ethics Review Committee of Jiangsu Provincial Centre for Disease Control and Prevention (JSJK2016-B003-03) and Zhongda Hospital, Southeast University. The patients/participants provided their written informed consent to participate in this study.

## Author contributions

HZ was responsible for epidemiological investigation. HG and TM were responsible for statistical analysis. YG, YW, and JY were responsible for guidance and supervision. All authors contributed to the article and approved the submitted version.
