# Corrigendum: A community-oriented survey on the association between androgenetic alopecia and metabolic syndrome in Chinese people

**DOI:** 10.3389/fmed.2022.1133463

**Published:** 2023-01-09

**Authors:** Hongliu Zhu, Haijian Guo, Yihong Gao, Yuegang Wei, Tao Mao, Jianqiu Yang

**Affiliations:** ^1^Department of Dermatology, Jiangyin Hospital of Traditional Chinese Medicine, Wuxi, China; ^2^Integrated Business Management Office, Jiangsu Provincial Center for Disease Control and Prevention, Nanjing, China; ^3^Department of Dermatology, The Affiliated Hospital of Nanjing University of Chinese Medicine, Nanjing, China; ^4^Department of Health Education, Jiangsu Provincial Center for Disease Control and Prevention, Nanjing, China

**Keywords:** androgenetic alopecia, metabolic syndrome, systolic blood pressure, dyslipidemia, diabetes mellitus

In the published article, there was a mistake in the Funding statement. The grant number for the National Natural Science Foundation of China was displayed as “Grant No. 51925203.” The correct Funding statement appears below.

